# Establishing and Validating a Biomolecular Signature of Ischemia/Reperfusion Injury in a Porcine Pancreas Allotransplantation Model

**DOI:** 10.1097/TXD.0000000000001793

**Published:** 2025-07-24

**Authors:** Samrat Ray, Sujani Ganesh, Laura Martinez-Arenas, Catherine Parmentier, Masataka Kawamura, Bhranavi Arulratnam, Christian Hobeika, Emmanuel Nogueira, Francisco Calderon Novoa, Markus Selzner, Trevor W. Reichman

**Affiliations:** 1 Ajmera Transplant Centre, Toronto General Hospital, University Health Network, Toronto, ON, Canada.; 2 Hepatology, Hepatobiliopancreatic Surgery and Transplant Laboratory, Instituto de Investigacion Sanitaria La Fe, Universitat Politecnica de Valencia, CIBEREHD, Valencia, Spain.; 3 Department of HPB Surgery and Liver Transplantation, Beaujon Hospital, AP-HP, Paris-Cité University, Clichy, France.; 4 Department of Surgery, University of Toronto, Toronto, ON, Canada.

## Abstract

**Background.:**

Despite considerable advancement in surgical and immunological management in pancreas transplantation, graft pancreatitis remains a feared complication after pancreas transplantation. Identification of molecular mechanisms of underlying ischemia/reperfusion injury (IRI) in pancreas transplantation could, therefore, pave the path for targeted therapy to improve surgical outcomes. The aim of the study was to identify and validate the genes differentially expressed in the early period (24 h) of graft reperfusion in pancreas transplantation.

**Methods.:**

A porcine pancreas allotransplant model (n = 4) was used to identify and validate the genes aberrantly expressed in 60 min postreperfusion tissue samples (phase 1). Trends of expression of selected genes from phase 1 and corresponding protein product levels in serum were validated at defined time points for >24 h in a technically replicated external cohort (n = 3; phase 2).

**Results.:**

A total of 104 genes were found to be upregulated at 60 min after pancreas graft reperfusion. The most consistently overexpressed genes were IL6, THBS1, and MIR-21 (micro-RNA) mapped to protein kinase and intracellular signaling molecular pathways. Levels of expression of these genes correlated significantly with serum interleukin-6 (*R* = 0.60–0.81; *P *< 0.01) and tumor necrosis factor-alpha levels (*R* = 0.34–41; *P* > 0.05) at corresponding time points.

**Conclusions.:**

The results provide new insights into biomolecular pathways (THBS1-IL6-MIR-21 crosstalk and hydroxymethylglutarate coenzyme A reductase–linked nuclear factor kappa B activation) linked to pancreatic IRI in porcine transplantation model. Identification and validation of some novel molecular pathway interactions in human pancreas transplantation could pave the path for potential targeted therapy in alleviating graft injury in the early phases of pancreatic IRI.

## Graphical abstract

**Figure:**
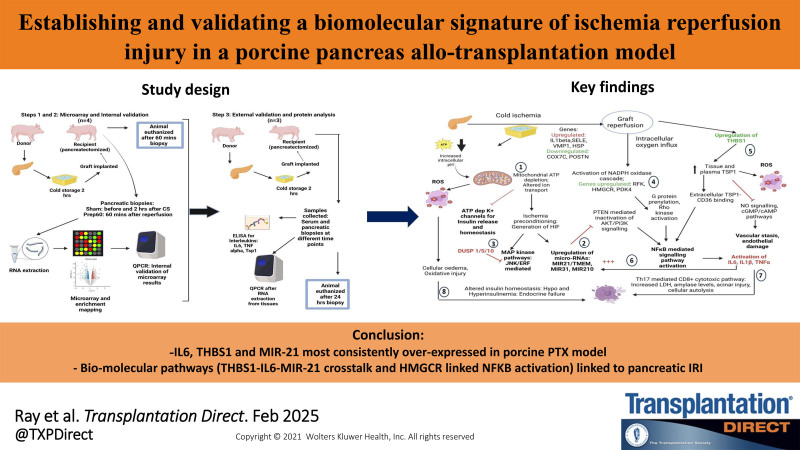

Over the past few decades, pancreas transplantation has shown promising results in extending and improving the quality of life of patients with diabetes mellitus and has resulted in the long-term cure of insulin-dependent diabetes mellitus.^1^ Although the graft survival rates after pancreas transplantation have improved during the past decades, long-term outcomes of pancreas transplantation are significantly worse compared with other whole organ allografts such as liver, kidney, and heart.^2,3^ Due to a combination of various factors, graft pancreatitis continues to be an ongoing issue in the early posttransplant setting. Immunological response and microcirculatory disturbances culminating into ischemia/reperfusion injury (IRI) play a major role in affecting the outcome in these patients.^4^ IRI represents the constellation of immunologic reactions that contribute to downstream effects of graft inflammation and damage after solid organ transplantation. Although there has been a large body of evidence in the literature regarding IRI and its effects in heart, kidney, and liver transplantation models,^5-7^ there is a paucity of literature in establishing the biomolecular and immunologic pathways implicated in pancreatic IRI. A few recent studies in murine transplantation models identified some genes upregulated in the warm and cold phases of IRI after pancreatic graft implantation.^8,9^ However, there still exists a lack of evidence to pinpoint the definitive molecular, biochemical, and immunological pathways leading to the downstream effects of IRI after pancreas transplantation, especially in the large animal or clinical allotransplantation models. Identifying and targeting potential genes and relevant biochemical pathways could be promising in alleviating the tissue injury associated with IR and resultant graft pancreatitis after transplantation and even offer potential targets for preconditioning grafts using normothermic machine perfusion, another area of potential interest in improving organ utilization rates in pancreas transplantation.

Therefore, we aimed to identify and validate the genes and their relevant protein products differentially expressed in the early period of graft reperfusion in a porcine pancreas allotransplantation model and formulate a biomolecular signature of IRI in pancreas transplantation. To our knowledge, this is a novel attempt at establishing a molecular and biochemical roadmap of IRI in porcine allotransplantation models.

## MATERIALS AND METHODS

### Study Protocol

**FIGURE 1.**
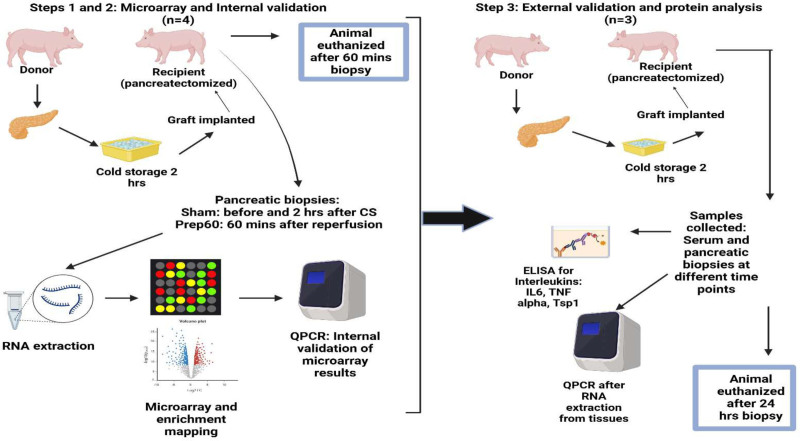
Summary of the study protocol. Steps 1 and 2 constituted phase 1 of the study protocol: a porcine pancreas allotransplantation model (n = 4) was used with a pancreatic graft recovered from a brain-dead donor animal. Minimal CS of 2 h in UW solution. Biopsies before and 60 min after reperfusion (Sham and Prep60, respectively). Step 3 constituted phase 2 of the study: external validation in a technical replicate of phase 1 (n = 3); 24-h survival allotransplantation model. Serum and pancreatic tissue biopsy samples before implantation (baseline) and 30 min, 60 min, 3 h, 6 h, and 24 h after implantation (postreperfusion). CS, cold storage; UW, University of Wisconsin.

The detailed study protocol is outlined in Figure 1. The study was conducted in 2 consecutive phases (phase 1: gene identification and internal validation and phase 2: external validation). A porcine pancreas allotransplantation model was used for both the phases. Briefly, the first phase (n = 4) involved graft pancreatectomy in the donor (heart beating donation) followed by 2 h of cold storage in a University of Wisconsin solution at 4 °C and implantation into a pancreatectomized recipient pig. Pancreatic wedge biopsies were obtained from the graft (pancreatic corpus) before cold storage (labeled as Sham 1, serving as control), after 2 h of cold storage (labeled as Sham 2, serving as the baseline), and 60 min after graft reperfusion (labeled as Prep60, serving as the main time point of interest). The animal was euthanized after the 60-min biopsy time point. The subsequent steps included RNA extraction, followed by microarray analysis, enrichment pathway mapping, and internal validation of the microarray results using quantitative reverse transcription polymerase chain reaction (RT-qPCR), details of which are outlined further. Phase 2 of the study involved pancreas allotransplantation using the same protocol as phase 1 in a new set of 3 experiments (n = 3; external validation cohort). The biopsies in this group were taken after 2 h of cold storage (baseline [BL]), 30 min postreperfusion (Prep30), 60 min postreperfusion (Prep60), 3 h postreperfusion (Prep3h), 6 h postreperfusion (Prep6h), and 24 h postreperfusion (Prep24h). Additionally, serum samples were also collected at these corresponding time points. The animals were euthanized after the 24-h tissue and serum samples were collected or earlier if deemed unfit based on humane grounds. The subsequent steps in phase 2 involved RNA extraction from the tissues, followed by RT-qPCR to validate the genes identified and validated in phase 1. Furthermore, ELISA was used to identify the trend of downstream protein products encoded by those genes and their proposed biochemical pathways in the serum samples at the respective time points. Surgery and RNA extraction from the tissues in both phases 1 and 2 were performed in sequence by the same surgeon (S.R.) throughout the study period.

### Animals and Surgical Procedure

The study was approved by the Animal Care Committee of the University Health Network Research Institute, ON, Canada (protocol ID: AUP 6245.1). Fifteen-week-old male Yorkshire pigs were used for the transplant model (35–40 kg). All animals received humane care and all procedures were performed in accordance with the “Principles of Laboratory Animal Care” and the “Guide for the Care of Laboratory Animals” published by the National Society for Medical Research and by the National Institutes of Health, respectively. The study was reported in compliance with the ARRIVE (Animal Research: Reporting of In Vivo Experiments) guidelines.^10^ The steps of donor pancreatectomy and recipient operation and postoperative surveillance were done according to the protocol reported by our group previously.^11,12^ Details of the same are available in **Surgery and Anaesthesia Protocol (Donor Operation**) and **Surgery and Anesthesia Protocol (Recipient Operation**) (**SDC**, http://links.lww.com/TXD/A754). The participating animals in both cohorts had a similar leucocyte antigen profile and were blood-compatible among the donor-recipient set.

### RNA Extraction and Microarray

For the experiments in phase 1 (n = 4), wedge biopsies were obtained from the pancreatic corpus at 3 time points (detailed previously) and placed in 1 mL RNAlater solution (Sigma-Aldrich, Oakville, ON) at 4 °C. RNA was extracted from the pancreatic tissue (n = 3 per experiment) using the RNeasy lipid mini tissue kit (Qiagen, Toronto, ON) as per manufacturer’s instruction, on the same day after the 60 min tissue biopsy and the quality was tested with a spectrophotometer. This was the most rate-limiting step of the study as extracting good quality RNA from an enzyme-rich tissue such as the pancreas was a formidable task, as also demonstrated previously by a few studies.^13,14^ The RNA samples (10–15 µL per Eppendorf) were stored at –80 °C, immediately after the extraction. RNA quality was confirmed using a Bioanalyzer 2100 with the RNA6000 Nano LapChip Kit (both Agilent Technologies Canada Inc, Mississauga, ON) at our collaborator’s facility (The Centre for Applied Genomics, SickKids, Toronto, ON). Samples were deemed adequate for microarray analysis based on the RNA integrity number (preferably ≥7) and/or the degradation assessment on gel electrophoresis by 18S and 28S ribosomal band patterns (more details available in **Figure S1, SDC,** http://links.lww.com/TXD/A754). The RNA extraction was performed for all cases by the same investigator (S.R.) using the same protocol to ensure the reliability of the technique and minimize the chances of RNA degradation due to contamination and laboratory handling. Transcriptome profiling of extracted RNA was performed using the GeneChip Porcine Gene 1.0 ST Array, examining a total of 8434 genes.

### Microarray Analysis

Microarray analysis was performed using the Transcriptome Analysis Console software (version 4.0.2, Thermo Fisher Scientific, Santa Clara, CA). The Transcriptome Analysis Console software combines the CEL and CHP file analyses and quality control in microarray analysis with a set of R and Bioconductor statistical modules such as Limma, EventPointer, and so on to provide a streamlined, user-friendly graphical interface for analyzing gene chips.^15^ Quality control was performed using the principal component analysis graphs (see **Figure S2, SDC,** http://links.lww.com/TXD/A754) based on signal intensities to compare the samples by sample attributes. After gene-level summarization using robust multiarray averaging^16^ with a variance cutoff of 0.35, the probes exhibiting little variability in expression across all samples were excluded. Differential expression testing was performed on the filtered data set (1834 probes) using the in-built eBayes method of ANOVA (*P* < 0.05, gene fold change <–2 or >2). The comparison groups in the analysis were Sham1 (control) versus Sham2 (baseline) versus Prep60 (main point of interest). Gene set filtering was further performed using the in-built Venn diagrams to identify the final genes of interest (119 gene probes).

### Gene Ontology and Enrichment Mapping

Gene ontology analysis for lists of differentially expressed genes from the above-mentioned step (119 gene probes) was conducted using g:Profiler.^17^ The expression set relating to the 2 time points of interest (Sham2 and Prep60) was input into the software and was filtered to include only the annotated genes and adjusted for significantly enriched (Benjamini-Hechberg false discovery rate-adjusted *P* value [*q*] < 0.05) gene ontology terms and pathway gene sets (including Kyoto Encyclopedia of Genes and Genomes, Reactome, and WiKiPathways). This step constituted the functional pathway analysis or the overrepresentation analysis. The EnrichmentMap plug-in of Cytoscape (version 3.9.1) was used to create an enrichment map of the g:Profiler results, depicting the overlap among pathways, with similar biological processes grouped together as subnetworks.^18^ Gene sets and pathways with a false discovery rate *P* value of <0.05 were used as input, and a conservative overlap coefficient (0.3) was used to build the enrichment map. Homologous human phenotype pathways were also mapped for the pathways identified by the enrichment mapping using the Metascape (**Figure S3, SDC,** http://links.lww.com/TXD/A754).^19^ Pathways of relevance and overlap were also sorted on the basis of the existing literature on IRI in other solid organs besides the pancreas.

### Internal Validation Using RT-qPCR

A total of 7 relevant genes were identified from the previously mentioned steps, based on the biochemical relevance, magnitude of fold change, and potential for therapeutic targeting of the pathways they encode. To ensure a better quality of RNA for RT-qPCR analysis (based on 260/280 ratio ≥2), the RNA samples from the previously mentioned time points were subjected to DNAse digestion using DNAse I-XT kit (New England BioLabs) before the PCR, followed by RNA clean-up (Monarch RNA clean-up kit, New England BioLabs). The complementary DNAs encoding interleukin-6 (*IL6* gene), thrombospondin-1 (*THBS1*), hydroxymethylglutarate coenzyme A reductase (*HMGCR*), micro-RNA-21 (*MIR-21*), dual-specificity phosphatase 5 (*DUSP5*), and riboflavin kinase (*RFK*), and cytochrome C oxidase subunit VIIc (*COX7C*) were amplified from the DNASe-treated RNA samples using Master mixes with the appropriate primer pairs (see **Table S1, SDC,** http://links.lww.com/TXD/A754). Quantitative PCRs were performed on an AB StepOnePlus Real-Time Polymerase Chain Reaction system (Applied Biosystems, Foster City, CA) at an annealing temperature of 56 °C. The relative abundance of a transcript was represented by the threshold cycle of amplification (CT), which is inversely correlated to the amount of target RNA/complementary DNA being amplified.^20^ The *GAPDH* gene was used as the housekeeping gene for the analysis. The delta CT values were calculated between the transcript of interest subtracted from the housekeeping gene for each experiment. Next, double delta CT values (delta CT of a gene of interest minus delta CT of control gene/baseline sample) were calculated for each experiment (n = 4). The relative quantitation or gene fold changes were expressed as 2^(-delta-delta CT)^.^21^

### External Validation of the Relevant Genes and Their Protein Products (Using RT-qPCR and ELISA)

The first step in the analysis involved RNA extraction from the pancreatic tissues retrieved at various time points from the 3 experiments in phase 2 and stored in RNA later solution, using the same kit and protocol as implemented for phase 1 of the study. The RNA samples were pretreated with DNAse I-XT and RT-qPCR was performed using the same protocol as mentioned previously (housekeeping gene: *GAPDH*). For this part of the analysis, we selected 3 genes (*IL6, MIR-21*, and *THBS1*) based on the following criteria: log2 gene fold change on RT-qPCR >2 or <–2 on RT-qPCR at 60 min postreperfusion time points (Prep60) and significant difference in expression with respect to Sham/baseline (*P* < 0.05; internal validation step), genes targeting biochemical and molecular pathways different from one another and genes with potential for pharmacologic targeting. The trend of gene fold changes at the various time points (baseline to 24 h postreperfusion) was plotted and compared between the 3 experiments. The second step in this phase involved validation of the downstream protein products upregulated or downregulated by these genes during IRI, using ELISA. The serum samples from the experiments at the designated time points (mentioned previously) were collected, snap frozen, and stored at –80 °C, to be used later for ELISA. Three important inflammatory mediators were identified from the pathway analysis of the previously mentioned genes: IL-6, tumor necrosis factor (TNF)-alpha, and thrombospondin-1 (TSP1), and their serum levels were studied at the time points corresponding to the tissue samples (baseline to 24 h). The gene fold changes were subsequently correlated to the levels of these inflammatory mediators.

### Statistical Analysis

All data were analyzed with GraphPad Prism software (version 10; GraphPad, San Diego, CA). The gene fold changes on RT-qPCR were expressed as log2 for comparison between Sham and Prep60 time points. Results were expressed as mean ± SD. The paired *t* test was used for comparison. The gene fold trend across the time points in the external validation cohort was compared using the mixed-effects analysis (2-way ANOVA). Gene fold changes and serum levels of inflammatory mediators at corresponding time points in the external validation cohort were also plotted against each other and correlated using Pearson’s correlation coefficient (*R*) subsequent to passing normality and log normality tests. *P* values of <0.05 were considered to indicate significance.

## RESULTS

### Genes and Biochemical Pathways Involved in the Early Phase of Pancreatic IRI (60 min Postreperfusion)

**FIGURE 2.**
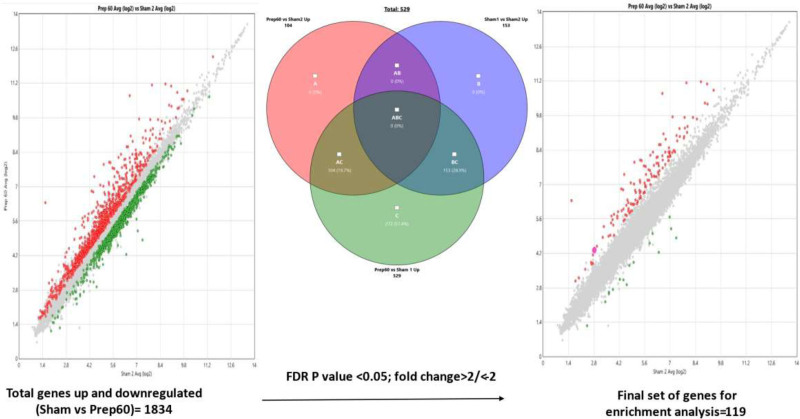
Summary of the total gene probes upregulated and downregulated by microarray analysis. Scatter plot showing the genes upregulated (in red) and downregulated (in green) at 60 min after reperfusion (represented by Prep60) relative to the baseline (represented by Sham2): x-axis representing log 2 gene fold change at Sham2 and y-axis representing log 2 gene fold change at Prep60. Genes showing altered expression at in pancreatic tissue after 2 h of cold storage relative to baseline shown by Sham1 (before cold storage) vs Sham2 (after 2 h of cold storage). Final genes for analysis after filtration using FDR *P* value of <0.05: 119 genes. FDR, false discovery rate.

**FIGURE 3.**
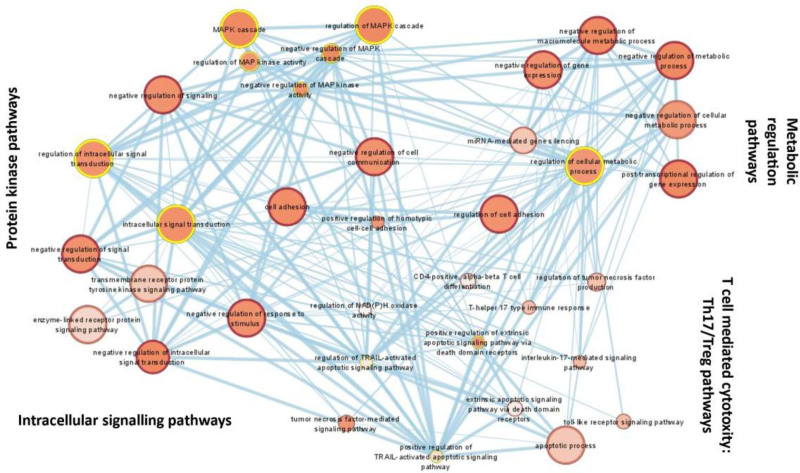
Key functional pathways identified on enrichment mapping. Enrichment mapping using Cytoscape: FDR *P* < 0.05 and overlap coefficient 0.3. Maximum gene transcript overlapping noted in the metabolic regulatory pathways (n = 25). FDR, false discovery rate; MAPK, mitogen-activated protein kinase; NADPH, nicotinamide adenine dinucleotide phosphate; Th17, T helper 17; Treg, regulatory T cell.

A total of 104 and 15 genes were found to be significantly upregulated and downregulated, respectively (detailed list in **Table S2, SDC,** http://links.lww.com/TXD/A754) at 60 min postreperfusion with respect to the baseline (after 2 h of cold storage: Sham2). The probes that lacked a gene assignment and remained annotated with transcript IDs (NCBI/RefSeq or Ensembl ID), sequence ID (GenBank), or genomic coordinates (GenScan) were filtered out from this set (n = 45 in the upregulated list and n = 7 in the downregulated list; Figure 2). Table 1 shows the summarized list of the genes (and their protein products) with altered expression at 60 min postreperfusion relative to the baseline. Further enrichment analysis mapped the relevant genes to 4 major molecular and biochemical pathways: (1) metabolic and gene profiling alteration pathways (maximum number of overlapping gene transcripts; n = 25), (2) protein kinase pathways, (3) T cell–mediated cytotoxicity and T helper 17–regulated pathways, and (4) intracellular signaling pathways (Figure 3). Overall, among the most consistently upregulated or downregulated and most abundant transcripts were those coding for regulation of metabolic processes and gene expression of mediators involved in flavin mononucleotide biosynthesis or nicotinamide adenine dinucleotide phosphate (NADPH) oxidase pathways and mitochondrial electron transport chain (upregulated: *ATF3*, *APOBEC1*, *IL6*, *HMGCR*, *THBS1*, *RFK*, *PDK4*, and *ZFP36*; downregulated: *COX7C* and *ZFP709* family) and those coding for intracellular signaling protein kinase pathways such as nuclear factor kappa B (NFκB) pathway (*IL6*, *MIR21*, *THBS1*, *FOS*, and *JUN*), mitogen-activated protein kinase signaling pathways (upregulated: *ATF3* and *DUSP1/5/10*), and the micro-RNA mediated protein kinase (PI3 kinase) pathways (upregulated: *MIR-21*; downregulated: *MIR-216-1* and *MIR-217-1*). Besides this, there was a consistent upregulation of genes encoding T helper 17–mediated cellular cytotoxicity pathways such as *IL6*, *THBS1*, *NFκBIZ*, and *ZFP36*, suggesting a downstream mechanism of apoptosis-mediated cellular injury and death. Based on the magnitude of gene fold change, consistency of altered expression across the identified molecular and biochemical pathways and potential for therapeutic targeting, 7 genes were identified for further validation: *IL6* (NFκB signaling, NADPH oxidase, and T helper 17–mediated pathways), *THBS1* (intracellular signaling/G protein kinase, NADPH oxidase, and NFκB signaling pathways), *MIR-21* (micro-RNA-mediated protein kinase signaling pathway), *HMGCR* (NADPH oxidase pathway), *RFK* (flavin mononucleotide biosynthesis and Kreb’s cycle regulation pathway), *DUSP5* (mitogen-activated protein kinase regulatory pathway), and *COX7C* (electron transport chain: cytochrome C oxidase pathway).

**TABLE 1.** Summary of the important porcine genes with consistently altered expression at 60 min after graft reperfusion (Prep60) relative to baseline, after 2 h of cold storage (Sham2)

Gene	Gene product	Log2-Fold change	FDR *P*	Functional pathway
*ATF3*	Activating transcription factor	20.43	0.00034	Metabolic pathway regulation
*MIR21*	Micro-RNA-21	8.48	0.00071	Intracellular signaling, protein kinase
*VMP1*	Vacuole membrane protein-1	8.48	0.00067	Cellular adhesion and intracellular signaling
*PDK4*	Pyruvate dehydrogenase kinase 4	7.74	0.00054	Metabolic pathway regulation
*FOS*	Leucine zipper proteins	7.7	0.00031	Intracellular signaling (with JUN), Metabolic pathway regulation, Protein kinase
*THBS1*	TSP-1	7.4	0.00034	Protein kinase, Metabolic pathway regulation, T cell–mediated pathways
*ZFP36*	Zinc finger protein 36	7.11	0.0051	T cell–mediated pathways, intracellular signaling
*IL6*	IL-6	4.87	0.00640	Protein kinase, Intracellular signaling, T cell–mediated pathways
*JUN*	Transcription factors	4.6	0.0032	Intracellular signaling (with FOS), metabolic pathway regulation
*ANKRD1*	Ankyrin repeat domain (transcription factor)	3.95	0.0061	T cell–mediated pathways, Metabolic pathway regulation (induced by IL-6 and TNF-alpha)
*SELE*	Selectin E	4.08	0.0051	Cellular adhesion and intracellular signaling
*NFκBIZ*	Nuclear factor for kappa B inhibitor zeta	3.38	0.0091	Protein kinase, intracellular signaling
*DUSP5* ^*b*^	Dual-specificity phosphatase 5	3.34	0.0065	Protein kinase (MAP kinase), intracellular signaling
*APOBEC1*	Apolipoprotein B mRNA editing enzyme catalytic subunit 1	2.9	0.0041	Metabolic pathway regulation
*HMGCR* ^*c*^	Hydroxymethylglutarate coenzyme A reductase (rate-limiting step in cholesterol biosynthesis)	2.9	0.00031	T cell–mediated pathways, intracellular signaling
*ZFAND5*	Zinc finger AN-1 type containing 5	2.68	0.0037	Metabolic pathway regulation
*RFK*	Riboflavin kinase	2.08	0.0041	Metabolic pathway regulation (FMN biosynthesis→ NADPH oxidase pathway)
*COX7C*	Cytochrome C oxidase subunit VIIc	–2.67	0.0051	Metabolic pathway regulation (electron transport chain in mitochondria)

^*a*^All the genes have been categorized into 4 functional pathways identified on enrichment mapping: metabolic pathway regulators, protein kinase pathway mediators, intracellular signaling mediators, and T cell–mediated pathway regulators.

^*b*^DUSP family (1/5/10) has counterregulatory effect on the MAP kinase pathway.

^*c*^HMGCR mediates the prenylation of G proteins by regulating cholesterol biosynthesis: key step in the generation of proinflammatory mediators.

FDR, false detection rate; FMN, flavin mononucleotide; IL-6, interleukin 6; MAP, mitogen-activated protein; NADPH, nicotinamide adenine dinucleotide phosphate hydrogen; TNF, tumor necrosis factor; TSP-1, thrombospondin-1.

### Internal Validation of the Microarray Findings

**FIGURE 4.**
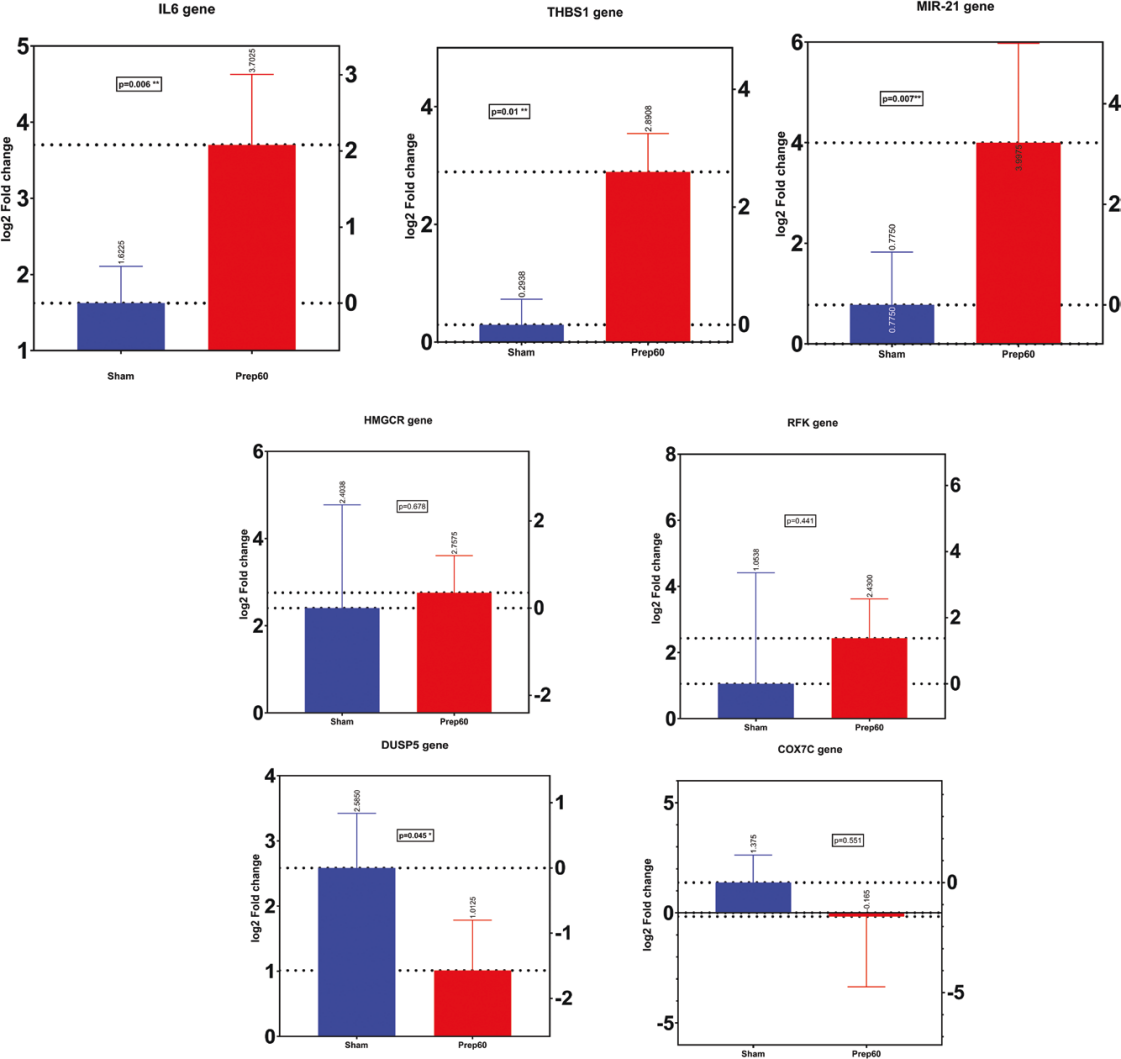
Internal validation of the selected genes with aberrant expression (upregulated or downregulated) at Prep60 vs Sham time points. Gene expression was assessed through RT-qPCR and relative to GAPDH (housekeeping gene). Expression is expressed as comparative CT of the gene of interest subtracted by the CT of the housekeeping gene for each sample (delta CT) with the mean value marked with a horizontal bar. Log2 fold change was >2 and significant (*P* < 0.05) at Prep60 (60 mins postreperfusion) time point relative to the baseline (Sham) for IL6, THBS1, and MIR21 genes. Log2 fold change difference at the 2 time points was significantly different for DUSP5 gene as well, but contrary to the microarray findings, the gene fold change was <2 at Prep60 time point (did not pass the internal validation). CT, threshold cycle for amplification; RT-qPCR, quantitative reverse transcriptase polymerase chain reaction.

Figure 4 summarizes the fold changes of the 7 selected genes detected on RT-qPCR at 60 min postreperfusion (Prep60) with respect to the baseline (Sham). Three of the 7 genes (*IL6, THBS1,* and *MIR-21*) exhibited a higher than 2-fold change in the RT-qPCR analysis at Prep60, with a significant difference from Sham (*P* < 0.05) and were subsequently selected for further validation in the external cohort (phase 2), based on this and the other criteria previously mentioned (see MATERIALS AND METHODS section).

### External Validation of the Genes and Protein Products

**FIGURE 5.**
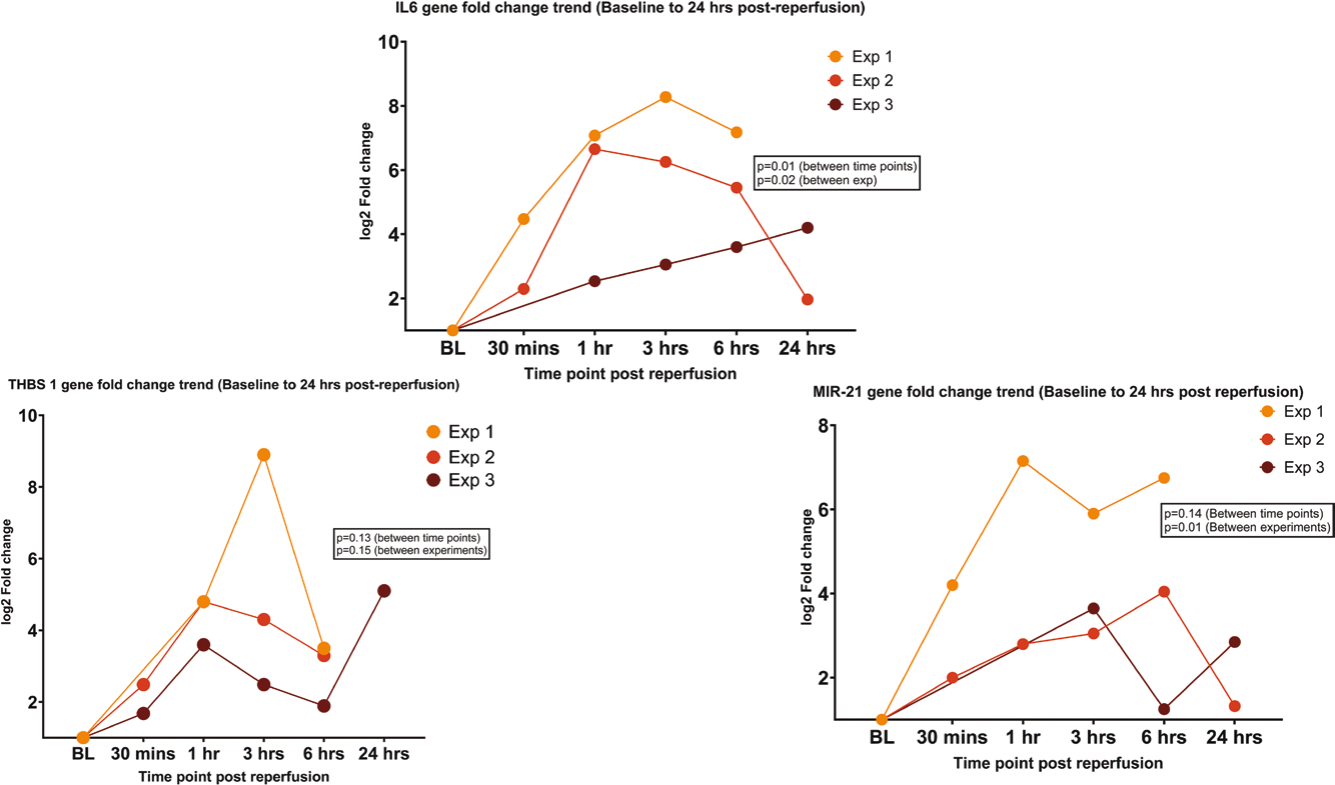
Trend of gene fold changes (IL6, THBS1, and MIR-21) at defined time points in the first 24 h after reperfusion for the external validation cohort (n = 3) compared. Comparison of the trend of log2 fold changes of IL6, THBS1, and MIR-21 genes at 30 min, 1 h, 3 h, 6 h, and 24 h postreperfusion with respect to the baseline between the 3 experiments in the external validation cohort (gene fold changes assessed using delta-delta CT method on RT-qPCR by SYBR green dye method). Note: the peak gene fold change was noted between 1 and 3 h for all 3 genes in the majority. Comparison between experiments and comparison between the time points for the individual genes was performed using the mixed-effects 2-way ANOVA (*P* < 0.05 significant). BL, baseline; Exp, experiment number; RT-qPCR, quantitative reverse transcriptase polymerase chain reaction.

**FIGURE 6.**
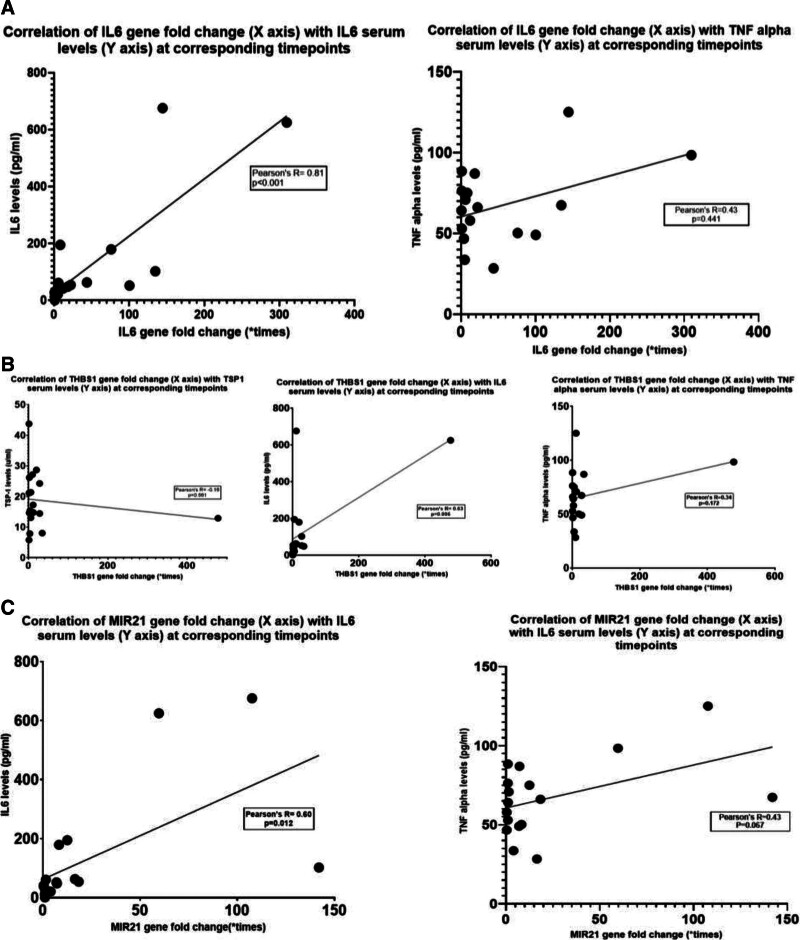
Correlation of the gene fold changes in pancreatic tissue with the relevant protein product levels in the serum at similar time points after reperfusion (baseline to 24 h postreperfusion): IL6 gene fold change against serum IL-6 and TNF-alpha levels (A); THBS1 gene fold change against serum IL-6, TNF-alpha, and TSP-1 levels (B); and MIR-21 gene fold changes against serum IL-6 and TNF-alpha levels (C). X-axis shows gene fold changes (expressed as 2^(-delta-delta CT)^ values) and y-axis shows serum levels of the protein product (unit/mL). Correlation using Pearson’s correlation coefficient (*R*); *P* < 0.05 significant. Data normality was assessed using the Kolmogorov-Smirnov test. IL, interleukin; MIR, micro RNA; TNF, tumor necrosis factor; TSP-1, thrombospondin-1.

Figure 5 illustrates the trend of gene fold changes across the defined time points in the 3 experiments of the external validation cohort. The maximum gene upregulation was noted for experiment 1, with a peak *IL6* and *THBS1* log2 fold change of >8 at 3 h postreperfusion and *MIR-21* (7.5-fold) at 1 h postreperfusion. This correlated with the clinical outcome of the transplantation in experiment 1, where the animal had to be euthanized at 10 h postreperfusion, in view of severe hemodynamic alterations and high inotropic support after graft implantation, displaying signs of severe IRI (persistently elevated lactate, fluid unresponsive acidosis, severe coagulopathy, and multiple parenchymal petechiae 3 h after reperfusion), not conducive to survival. The trend of *IL6* and *MIR-21* gene fold changes were significantly different between the 3 experiments across the different time points (*P* = 0.02 and *P* = 0.01, respectively). Further correlation of the gene fold changes with the levels of downstream protein product mediators (IL-6, TNF-alpha, and TSP-1) revealed a strongly positive correlation of serum IL-6 levels with *IL6* (*R* = 0.81; *P* < 0.001), *THBS1* (*R* = 0.63; *P* = 0.006), and *MIR-21* (*R* = 0.61; *P* = 0.016) gene fold changes. Although there was a positive correlation of TNF-alpha levels with *IL6* (*R* = 0.43), *THBS1* (*R *= 0.34), and *MIR-21* (*R* = 0.43) gene fold changes, this was not found to be significant (Figure 6). TSP-1 levels showed a negative correlation with the *THBS1* gene fold change, although this was not found to be significant (*R*= –0.16; *P* = 0.561). The levels of serum amylase and lactate dehydrogenase levels (markers of cellular injury) showed no significant correlation with the IL-6 and TNF-alpha levels at the corresponding time points (**Figure S4, SDC,** http://links.lww.com/TXD/A754).

## DISCUSSION

**FIGURE 7.**
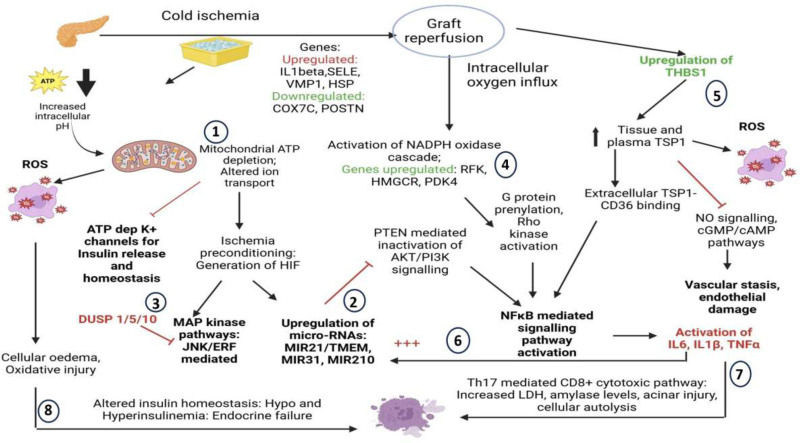
Summary of the proposed pathophysiology of pancreatic IRI in porcine pancreas allotransplantation, depicting the interaction of the genes upregulated and downregulated with the functional pathways identified and the downstream effect in the early phase (first 24 h) after pancreas transplantation. Proposed pathway of interaction: (1) mitochondrial ATP depletion and altered electron transport chain in the cold ischemic phase (COX7C/POSTN downregulation) → inhibition of ATP dependent insulin release from islet cells; In response to ischemia: generation of HIF → ischemic preconditioning phase → (**2**) upregulation of micro-RNA pathway (MIR-21) causing inhibition of PTEN-mediated inactivation of PI3K-AKT signaling → counter-activation of NFκB-mediated signaling pathway and (**3**) MAP kinase pathway activation (counter-regulation by DUSP 1/5/10 genes); in response to reperfusion: influx of oxygen in the intracellular compartment → (**4**) upregulation of NADPH oxidase pathway of free radical injury (mediated by HMGCR and RFK metabolic genes) → ρ kinase pathway activation; (**5**) upregulation of THBS1 gene → extracellular binding of TSP-1 with CD36 and inhibition of nitric oxide/cGMP kinase pathways (endothelial damage and vascular stasis) → (**6**) activation of NF kappa B pathway (main mediator acute inflammatory cascade) → release of IL-6, TNF-alpha, IL-1β → inflammosome mitochondrial cascade (counter-activation of IL-6 release by IL-1β and TNF-alpha); counter-activation of micro-RNA pathway (**2**) and (**7**) Th17-mediated CD8^+^ cytotoxicity → activation of proapoptototic mediators (TNF-alpha mediated/Fas-CD95 mediated) → cellular lysis and death. Through activation and release of ROS → (**8**) cellular oxidative injury. Persistent cascade of this biomolecular interaction (accentuated in high-risk marginal grafts) → (**8**) Islet cell damage → altered insulin homeostasis → graft failure. Activation is shown by black arrows and inhibition is shown by red lines. cAMP, cyclic AMP; cGMP, cyclic GMP; ERF, ETS2 repressor factor; HIF, hypoxia inducible factor; IL, interleukin; JNK, Janus kinase; LDH, lactate dehydrogenase; MAP, mitogen-activated protein; NADPH, nicotinamide adenine dinucleotide phosphate; PTEN, phosphatase and tensin homolog deleted on chromosome 10; ROS, reactive oxygen species; Th17, T helper 17; TNF, tumor necrosis factor; TSP-1, thrombospondin-1.

Despite the high susceptibility of pancreatic grafts to IRI and its downstream detrimental effects on the clinical outcome after pancreas transplantation, few studies have addressed the events surrounding this initial inflammatory insult at a biomolecular level. One of the earliest pieces of evidence in the literature comes from the work by Benz et al^8^, who identified a complex interplay of proinflammatory genes associated with the transcription factors NFκB, activator protein-1, early growth response gene-1 (*EGR*-1), and zinc finger protein-9 in the early phase of pancreatic warm IRI in murine models. This was followed by Drognitz et al^9^, who identified a total 49 genes in a murine model of pancreas transplantation with consistent upregulation of more than 3-fold in all groups of varying cold ischemia time ranging between 6 and 18 h of storage in a University of Wisconsin solution or physiologic saline solution, before transplantation. Prominent genes identified by this group included transcription factors, cytoskeletal factors, heat-shock proteins (*HSP27* and *HSP90*), inflammatory mediators (*PAPIII*), and certain genes (*BEST5*) that had not been associated with IRI so far in any other solid organ transplantation model. Our results identified 3 important biomolecular pathways to be the key mediators of the downstream effects of IRI after porcine pancreas allotransplantation with minimal cold ischemia time of 2 h: NFκB) pathway (activated by *IL6* gene, the interplay of *IL6* with *MIR21* gene and by *THBS1* gene), phosphatidylinositol-3 kinase (PI-3K)-Akt signaling pathway (*MIR-21* regulated), and Th17-mediated cytotoxic pathway (*IL6*, *THBS1*, and *MIR-21* mediated). Based on our findings, we proposed a schematic interaction of the genes-pathways-protein products in an early phase of pancreatic IRI, depicted in Figure 7.

IL6 gene encodes for the proinflammatory cytokine IL-6, which has been implicated as a pleiotropic mediator of a plethora of systemic immunological and inflammatory responses and disease processes.^22^ Besides this, IL-6 has also been found to contribute to the activation of the coagulation system in experimental models of endotoxemia due to the existence of crosstalk between inflammation and coagulation, furthering the tissue damage in cardiac IRI models.^23^ IL-6 works in concert with IL-1β and TNF-alpha as a key mediator of the mitochondrial inflammasome pathway (positive feedback loop of NFκB activation and IL-6-mediated damage-associated molecular patterns release)^24^ and micro-RNA-mediated inhibition of phosphatase and tensin homolog deleted on chromosome 10 (*PTEN*) regulated PI3 kinase-Akt signaling inhibition) to cause further activation of NFκB and downstream signal transducer and activator of transcription (STAT) signaling pathways of inflammation in the early phase of graft injury.^25^ In the delayed phases of IRI, IL6 has been proposed to be protective after liver transplantation in murine models by activation of antiapoptotic mediators such as Bcl2, Bcl-xl, and cytoprotective heat-shock proteins.^26^ Besides a good correlation of serum IL-6 levels with the *IL6, THBS1*, and *MIR-21* gene fold levels at various time points after reperfusion (till 24 h), we also found IL-6 levels to be more consistently related to the clinical outcome of the animals in the experimental model of transplantation. The serum levels of TNF-alpha although it showed a corresponding change in trend in the first 24 h with the gene fold changes, did not reach a level of statistical significance in terms of correlation. This could indicate the possible dominance of TNF-alpha in pathways relating to cytotoxicity-mediated cellular death after 24–48 h of reperfusion and a paracrine mechanism of TNF in mediating the cellular cytotoxicity pathways, not necessarily reflected in the corresponding serum levels; however, this would need to be validated at those time points, in future studies.

MIRs are endogenous (18–22 nucleotides) RNA molecules with a ubiquitous role in regulating gene expression.^27^ The gene encoding pri-MIR-21 is mapped to human chromosome 17 of the *TMEM49* gene.^27^ Emerging evidence from the literature suggests the possible mechanism of *MIR-21* in renal IRI, with both protective and pathologic roles through multiple pathway regulation.^28^ *MIR-21* has been shown to affect the kinase signaling pathways (NFκB/STAT) through its counter-regulatory effect on the *PTEN*-mediated inhibition of the PI-3K-Akt signaling.^29^ The resultant effect is the activation of the NFκB/STAT pathways with increased expression of proinflammatory mediators such as IL-6 and TNF-alpha (inflammatory cascade). Overexpression of *MIR-21* in association with T helper cell differentiation, mediating regulatory T cell–related counter-regulation of immune cells in inflammatory processes has also been demonstrated, suggesting its possible protective role.^30^ Based on our findings, we suggest a possible role of MIR-21 in the early phase of pancreatic IRI by counter-regulation of *PTEN*-mediated PI3 kinase pathway inhibition, causing downstream upregulation of the NFκB pathway and IL-6 and TNF-alpha release (as evidenced by strong correlation of *MIR21* gene fold change with IL6 levels, *r* = 0.61, *P* = 0.016 and with TNF-alpha; *r* = 0.43; *P* = 0.06).

Another potential candidate gene was the *THBS1*, encoding for the protein product TSP-1. TSP-1 is an extracellular matrix glycoprotein, interacting with the membrane-bound proteins and proteoglycans such as integrins and lipoprotein receptor–related protein-1 to activate the downstream protease pathways, thereby mediating chemotaxis, cellular adhesion, angiogenesis, and cellular motility.^31^ Extracellular binding of TSP-1 with CD36 in response to acute inflammation has been shown to mediate activation of the NFκB pathway, leading to overexpression of TNF-alpha and IL-6/STAT proinflammatory pathways with downstream effects of acute tissue inflammation and injury.^32^ This interaction has also been shown to activate the CD47-dependent pathways, leading to the generation of apoptotic signals through the Fas/CD95-dependent cellular cytotoxicity cascade, causing resolution of inflammation and tissue death.^32^ Inhibition of nitric oxide signaling (cyclic AMP and cyclic GMP mediated) by TSP-1 has also been shown to cause vascular stasis and endothelial damage, both of which are hallmark features of IRI.^33^ This has been further substantiated by Yao et al^34^, who demonstrated the role of TSP-1 in accentuating O2-free radical-mediated damage in renal IRI models by phosphorylation of the Signal regulatory protein-1 (SIRP1) in nonphagocytic cells.^34^ Recent evidence from the cardiac IRI models has also identified the role of *THBS1*/TSP-1 in mediating mitochondrial fission, thereby exacerbating reactive oxygen species–mediated oxidative damage in aging myocardial tissues.^35^ An interesting finding in our study was a negative correlation (not statistically significant) of the *THBS1* gene fold change with the serum TSP-1 levels but a positive correlation with the serum IL-6 and TNF-alpha levels at the same time points. This could possibly be explained by an upregulation of the *THBS1* gene (in response to pancreatic graft reperfusion) leading to increased TSP-1 mediated NFκB pathway activation with an increase in IL-6 and TNF-alpha levels in the extracellular space causing the inflammatory graft response, subsequently resulting in “exhaustive consumption” of serum TSP-1 levels by the TSP-1/CD36 extracellular receptor interaction driving the inflammatory circuit. Besides this, activation of counter-immune regulatory pathways (transforming growth factor-beta and Fas/CD95-mediated apoptosis) by the TSP-1/CD36 interaction could also contribute to a negative feedback regulation of the serum TSP-1 levels over time in pancreatic IRI.

Besides these key biomolecular pathways, our study also highlighted some other important genes, with potential for therapeutic targeting in pancreatic IRI, that are worth mentioning. One of them is *HMGCR,* encoding the rate-limiting enzyme in cholesterol biosynthesis. HMGCR-mediated prenylation of G protein/ρ kinase pathway contributes to downstream activation of NFκB-IL6 cascade, inhibition of which could ameliorate proinflammatory cascade activation, as shown in experimental murine renal IRI models.^36,37^ Competitive inhibition of the HMGCR enzyme by statins is well-established therapy in hyperlipidemias, and it has also been shown to alleviate the inflammatory injury associated with atherosclerosis by the mechanism described earlier.^36^ Ease of administration and wide inexpensive availability of this class of drugs could be an attractive option to reduce inflammatory damage in pancreatic IRI if validated in future studies.

The strength of this study lies in being the pilot attempt at deciphering the molecular and biochemical mediators of pancreatic IRI in a large animal model, with further validation in a similar cohort of allotransplantation 24-h survival model. A major limitation of the study lies in the difficulty of technical replicability of the experimental design. This is mainly attributed to the challenging task of extracting consistently good quality RNA with minimal ribosomal degradation, which is because of the very high RNAse content in pancreatic tissue, aggravated further during reperfusion, thereby making the RNA quality very unpredictable for microarray analysis. This was further accentuated by the DNA contamination of these samples, making us add the step of DNAse pretreatment and RNA clean-up before RT-qPCR during internal and external validation to ensure higher reliability of the CT and relative quantitation values. We factored in this confounder while analyzing the findings and discrepancies between the microarray and RT-qPCR results in the phase 1 cohort. Besides this, the technical challenges associated with the porcine transplant surgery in a survival model and retrieving biopsies from a highly sensitive pancreatic graft at multiple time points postreperfusion added further to the difficulties of the model.

Another limitation of the study lies in the lack of a concrete and specific linking of the gene fold changes to the corresponding changes in the levels of downstream protein products. This is mainly attributed to the association of many of these protein products, especially IL-6 and TNF-alpha, with most acute inflammatory systemic cascades, thereby making their specific association with pancreatic IRI difficult to conclude. This would need further validation in a more heterogeneous model of pancreas transplantation in the future. Besides this, although these biomolecular markers provide significant cues in constructing a roadmap of pancreatic IRI in the first 24–48 h of transplantation, none of them should be construed as being markers of the endocrine and exocrine outcomes of the pancreatic graft, which are determined by a combination of several other factors. Alleviating the detrimental effects caused by IRI, including graft pancreatitis, vascular thrombosis, and graft edema, can reduce the risk of early graft failure. However, immunologic crosstalk with CD4^+^ and CD8^+^ T cell–mediated allograft rejection pathways (pathways distinct from IRI)^38,39^ could play an additive effect on the outcomes of IRI and need to be factored in, especially for therapeutic targeting of the IRI genes and their products. Currently, there are no definitive therapeutic targets validated to address the pathways of IRI in any solid organ transplantation. Etanercept, an inhibitor of TNF-alpha receptor, has been shown to ameliorate myocardial IRI by upregulation of cardioprotective adiponectin in murine models.^40^ However, the CTOT-19 (Clinical Trials in Organ Transplantation) trial in deceased donor renal transplantation model using induction with infliximab (anti-TNF-alpha) did not show any benefit and was associated with increased BK virus infection in the recipients.^41^ IL-6 receptor blockade with tocilizumab, in murine models, has similarly been shown to demonstrate decreased serum levels of IL6, TNF-alpha, and oxidative injury mediators such as malondialdehyde in renal IRI.^42^ Most of these trials have focused on targeting the protein products, with very limited evidence on targeting the genes to modulate the downstream pathway in IRI in solid organ transplant models.

In conclusion, the phenomenon of ischemia/reperfusion results in significant pancreatic parenchymal injury associated with the upregulation of various genes and the resultant production of proinflammatory cytokines and other mediators, leading to a vicious cycle of IRI. The findings of this study, along with the existing evidence from the literature, provide an insight into the possibility of identifying and targeting the genes associated with pancreatic IRI, with a future goal of reducing the acute cellular injury in the early period of graft reperfusion besides improving the pancreatic graft physiology and hemodynamics, when subjected to normothermic machine perfusion^43,44^ (an area of evolving interest to optimize graft utilization rates in pancreas transplantation). These findings highlight the framework by which biomolecular mechanisms of pancreatic IRI may be understood, need to be validated further in a more heterogeneous set of experimental models, and pave the path for identifying novel therapeutic targets to ameliorate the early effects of pancreatic IRI in the future.

## ACKNOWLEDGMENTS

The study was conducted in collaboration with the microarray facility at The Centre for Applied Genomics (TCAG), SickKids, Toronto, ON. The authors thank Dr Chao Lu, Xiaolin Wang, and Lan He from the TCAG facility for their efforts in carrying out the microarrays for the phase 1 of the study (2022–2023).

## Supplementary Material


