# Supplementary material for: Establishing and Validating a Biomolecular Signature of Ischemia/Reperfusion Injury in a Porcine Pancreas Allotransplantation Model
Source: Transplant Direct. 2025 Jul 24;11(8):e1793. doi: 10.1097/TXD.0000000000001793 (PMC12289139; doi:10.1097/TXD.0000000000001793)
Supplement: Supplementary file 1 [file txd-11-e1793-s001.pdf]

Establishing and validating a biomolecular signature of ischemia reperfusion injury in a porcine pancreas allo-transplantation model

Supplementary content

Surgery and anaesthesia protocol (Donor operation)

A policy of *minimal handling of the native pancreas* was followed to ensure a minimally inflamed graft at the end of the operation. After systemic administration of heparin (500 IU/kg BW) through the central venous catheter, blood was drained. This was followed by cross clamping of the supra-hepatic abdominal aorta and injection of Potassium chloride (10 ml) to induce cardiac death (*Minimum warm ischemia*). Immediately after this, cold flushing with 1L Ringer lactate (primed with 10000 IU Heparin), followed by 1L of UW solution (primed with 10000 IU Heparin) and venting out of the blood through a lateral opening in the portal vein and infra-hepatic inferior vena cava (IVC) were performed. This was followed by dissection of the graft en masse with the spleen, surrounding fasciae and retroperitoneal tissues and intact aorta, taking care to preserve the coeliac trunk and the superior mesenteric branches. The graft was subsequently flushed with 500 ml of UW solution and stored in an iced organ box at 4 degrees Celsius for 2 hrs.

Surgery and anaesthesia protocol (Recipient operation)

Broad spectrum antibiotics (Metronidazole 500 mg and Cefazolin 1 gm) and Proton pump inhibitor (Pantoprazole 20 mg IV) were administered at induction. The surgery comprised of two parts: Pancreatectomy and Graft implantation. Surgery started by fascial dissection along the groove between pancreatic head (duodenal lobe), Duodenojejunal flexure and the transverse

colon. The duodenal lobe was mobilised from the underlying main portal vein trunk and the pancreatic tail (splenic lobe) mobilised from the underlying splenic vein and spleno-portal junction. The pancreatic duct was identified, ligated and divided and the pancreatectomy completed by removing the native pancreas en masse. After removing the graft from ice, the recipient IVC was partially occluded using the side biting Satinsky clamp and venous anastomosis performed with the graft portal vein in an end-side continuous fashion. This was followed by arterial anastomosis between the graft aorta (proximal end) and the recipient aorta in an end-side continuous fashion using the Parachute technique. After securing an adequate hemostasis with Tranexamic acid and stabilising hemodynamics by adjusting the inotropic infusion, bowel anastomosis was performed in a side-to-side continuous fashion between the graft duodenum and recipient jejunal loop. Abdominal closure was performed in two layers (rectus sheath followed by skin) in a continuous fashion. The recipients in the phase 1 cohort were euthanized after 60 mins of reperfusion (time point of interest). For the recipients in the external validation cohort (Phase 2) targeted for 24 hrs survival, the central venous catheter was secured in the neck by creating a subcutaneous tunnel and used later for sample collection and drug administration. The animal was transferred to the housing facility, extubated and monitored for the next 24 hours. Post-operatively, the animals received intravenous (iv) Cefazolin 1 gm twice a day and iv Buprenorphine for pain control till euthanasia, besides the appropriate iv fluids.

Figure S1: Qualitative assessment of pancreatic RNA by Bioanalyser (Bioanalyzer 2100 with the RNA6000 Nano LapChip Kit, Agilient)

9 samples per experiment were sent: 3 samples for each time point : Sham1, Sham2 and Prep60. The sample with the most preserved 18S and 28S ribosomal bands on chromatography plate was

selected for microarray analysis. The chromatographic pictures of the samples for each experiment are shown below (from B1 to B2/Left to right: Sham1 to Prep60, with 3 samples in each time point). The arrow heads in the chromatography point at the 18S and 28S ribosomal bands. A1 represents the electronic ladder control.

Exp 1:

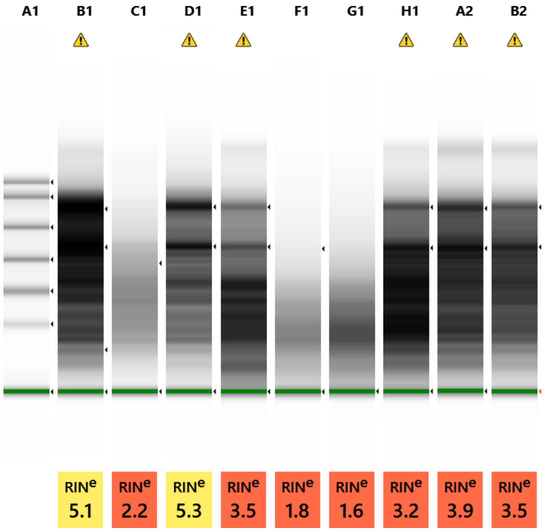

Exp 2:

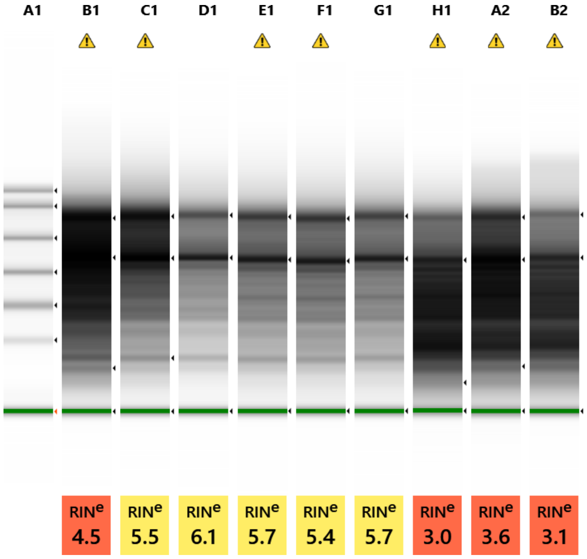

Exp 3:

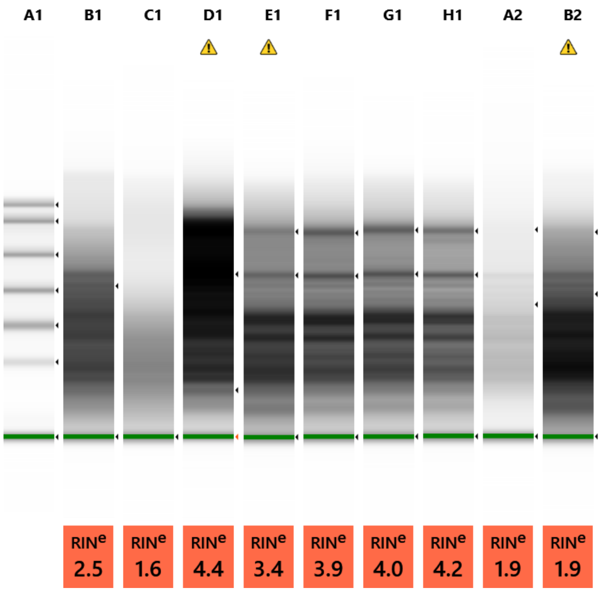

Exp 4:

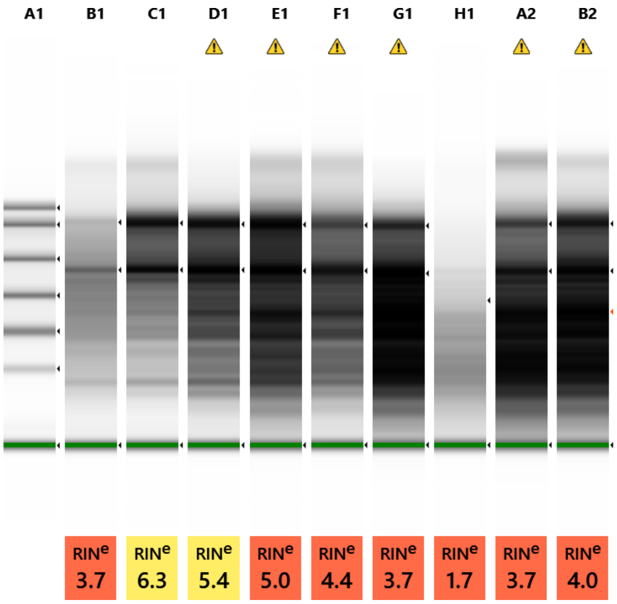

***Footnote: RIN: RNA integrity number (marker of relative degradation of RNA; an indicator of RNA quality). Range of RIN: 0-10 (10 been the least degraded). Factors like contamination with DNA in the sample also accounted for lower RIN values, besides inherently rich RNAse content in pancreatic tissues. Besides RIN, the 18S and 28S ribosomal band intactness on the chromatography were accounted for, while selecting the samples for microarray analysis.*

Figure S2: Principal component analysis (PCA) plots

QC was carried out using the principal component analysis (PCA) graphs based on signal intensities to compare the samples by sample attributes: Blue: Sham1, Red: Sham2 (Baseline), Purple: Prep60 (point of interest).

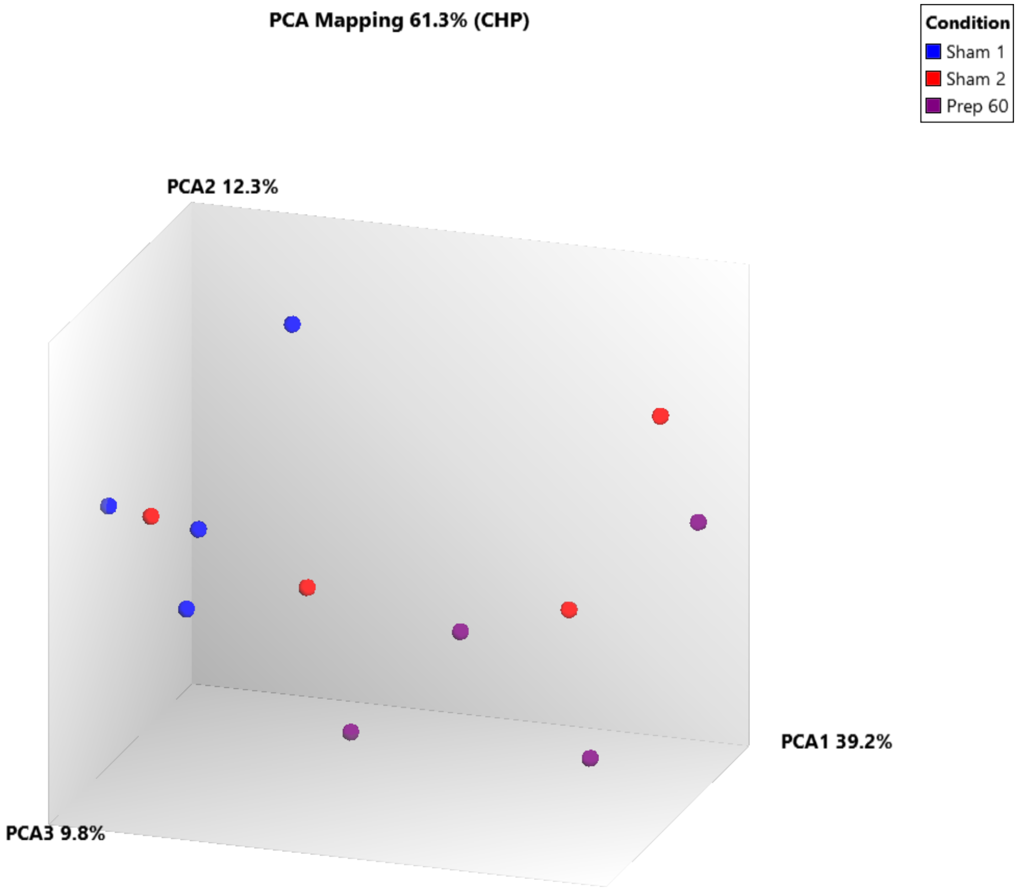

***Footnote: PCA % denotes the degree of similarity (overlap) between the observations in the 3 groups compared*

Figure S3: Homologous human phenotype pathways for the pathways mapped using enrichment mapping of the porcine genes (Metascape)

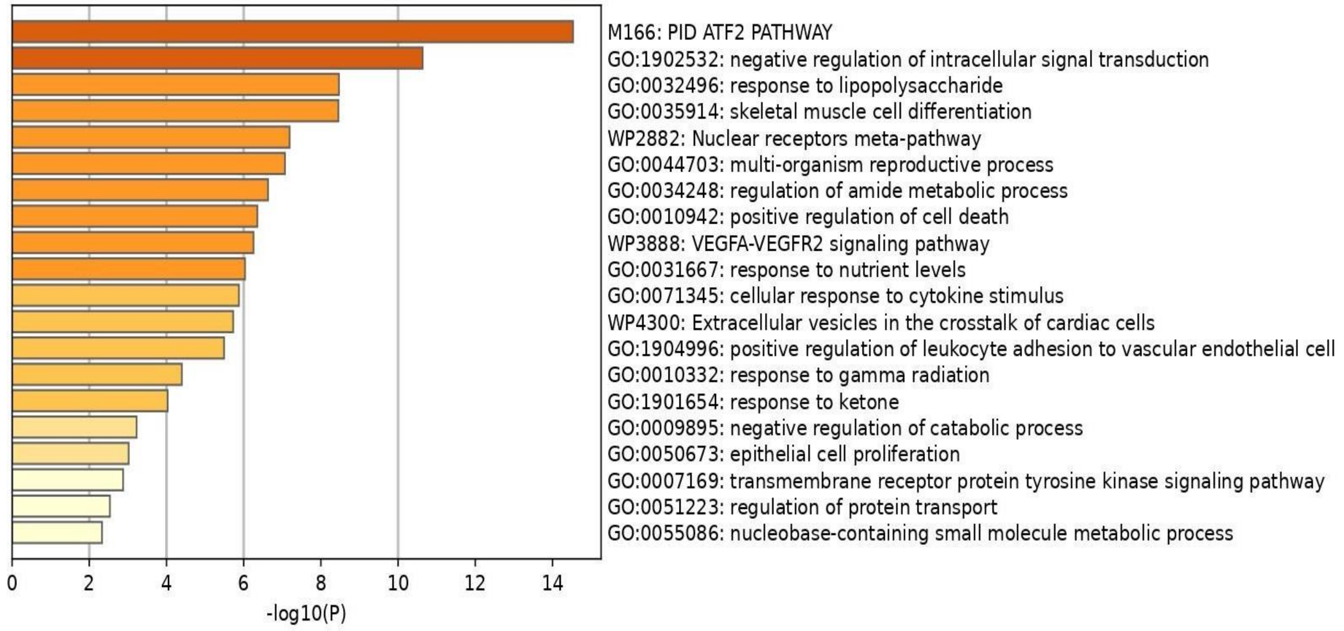

Table S1: Forward and reverse primer sequences for the genes of interest

Gene	Primer sequence	Tm
**IL6**		
Forward	5’- CAATCTGGGTTCAATCAGGAGA	62.0°C
Reverse	5’- TGCACAGCCTCGACATTT	62.0°C
**HMGCR**		
Forward	5’-CTGCTGCTGTAAACTGGATAGA	61.9°C
Reverse	5’-CTCAACCATCGCTTCTGTAGT	61.8°C
**THBS1**		
Forward	5’- CCGCAAAGTGACTGAAGAGA	62.0°C
Reverse	5’- CACTGTCCACTCCTCGTTATTC	62.1°C
**MIR21**		
Forward	5’- CACCTTGTCGGGTAGCTTATC	62.0°C
Reverse	5’- AATGTCAGACAGCCCATCG	61.9°C
**RFK**		
Forward	5’- CCATAAGATGGTGGTGAGCATAG	62.4°C
Reverse	5’- CCCATAGAAGTCCTCTTTGAAGG	62.1°C
**DUSP5**		

Forward	5'- TCATCAGTCAGTGTGGGAAAC	62.0°C
Reverse	5'- GGTAGAGGAACGGAAGGATTTC	62.0°C
**COX7C**		
Forward	5'-CGACATGTTGGGACAGAGTATT	62.1°C
Reverse	5'-AGCTAGTAACCGCCACTTATTT	61.9°C

***IL6: Interleukin 6, HMGCR: Hydroxymethylglutarate co-enzyme A reductase, THBS1: Thrombospondin 1, MIR21: Micro-RNA 21, RFK: Riboflavin kinase, DUSP5: Dual specific phosphatase 5, COX7C: Cytochrome C Oxidase subunit VIIc*

Table S2: Total genes up- and down-regulated in the early phase (60 mins) of reperfusion of porcine pancreatic allotransplantation graft with respect to the baseline

Gene ID	Gene	Fold change
15320854	LOC102157763	27.96
15332643	ATF3	20.43
15266898		11.17
15208468	MIR21; VMP1	8.48
15320856	CXCL2	8.18
15331363	PDK4	7.74
15312975	FOS	7.7
15287934	LOC396594	7.68
15195370	THBS1	7.4
15243247	DUSP1	7.21
15228898	ZFP36	7.11
15349031		6.75
15222862	RCAN1	6.6
15229317	NFIL3	6.51
15339245		5.72
15252885	LSMEM1	5.69
15327424	IL-6; IL6	4.87
15300578	JUN; LOC106508708	4.6
15291749	RND1	4.59
15326827	BTG2	4.58
15349061		4.55
15259635	EGR1	4.46
15339471		4.43
15201579	RGS2	4.4
15349063		4.39
15350995		4.39

15351131		4.32
15291441	MAFF	4.23
15254582	LOC100621891	4.22
15344185		4.13
15279419	SELE	4.08
15233409	ANKRD1	3.95
15237143	COQ10B	3.86
15340135		3.64
15218292	NFKBIZ	3.38
15286982	CYR61	3.38
15218765	ETS2	3.36
15228428	DUSP5	3.34
15244214		3.26
15336354	MIR222	3.24
15198190	KLF4	3.17
15306230	DNAJB4	3.17
15292014	LOC100525396; LOC100525528	3.14
15323275	LOC100525396; LOC100525528; LOC100620997	3.14
15343473		3.13
15341953		3.11
15223003	CKS2	3.08
15229543	STC1	3.04
15336352	MIR221	3.02
15217100	TSC22D2	2.99
15293831	APOBEC1	2.99
15350337		2.92
15258391	HMGCR	2.9
15340375		2.89
15192811	GJA1	2.87
15264711	DNAJB1; LOC100627777	2.85
15232407	EGR2	2.83
15304782	EPHA2	2.82
15303123	PLAUR	2.81
15279696	ADAMTS4	2.79
15265731	ENC1	2.78
15237118	NABP1	2.72
15204824	HSPH1	2.7
15189172	ZFAND5; LOC100737314	2.68
15267528	LOC100522543	2.64
15275046	SLC20A1	2.62
15188774	PLIN2; LOC100738250	2.61
15324478	SPP1	2.57
15242681		2.56

15309380	CDKN1A; LOC100623143	2.55
15209828	NDEL1; LOC100739126	2.54
15282785	SQLE	2.51
15239499		2.48
15247587	PARD6B	2.48
15347633		2.46
15351119		2.42
15222723	SAMSN1	2.41
15346039		2.37
15222609	LOC100157058; FILIP1L	2.36
15293768		2.36
15347035		2.36
15221578	TM4SF1	2.31
15283238	KLF10; LOC100736716	2.28
15283248	KLF10; LOC100736716	2.28
15345419		2.28
15190797	SLC25A25	2.26
15287672	CRY1	2.25
15283582	OSGIN2	2.24
15347637		2.22
15347635		2.21
15268996	DCUN1D3	2.2
15276635	ZFP36L2	2.19
15308333		2.19
15347639		2.18
15345409		2.14
15201790	DUSP10	2.12
15339053		2.12
15301930		2.1
15347031		2.09
15197627	RFK	2.08
15325306	PCF11	2.08
15345411		2.08
15221937	LIPH	2.04
15293676	EMP1	2.02
15314374	LOC100152126	-2.14
15312428		-2.54
15233436		-2.55
15258735	COX7C	-2.67
15283530	LOC100737436	-2.87
15264331	LOC100520720	-2.98
15257072	LOC100517213	-3.21
15350619		-3.36

15234522		-4.54
15314402		-5.21
15271394	MIR217-1	-7.96
15276376	MIR217-1	-7.96
15271392	MIR216-1	-8.3
15276378	MIR216-1	-8.3
15271390		-8.37
15183680		
15184480	PNRC1	
15184942		
15189179	ANXA1	
15192465		
15194997	LOC106509016	
15195272	RAD51	
15196326		
15202399		
15204149	KLF5	
15204255	MIR19B-1	
15204257	MIR92A-1	
15212667	CCL2	
15215070	ABHD5	
15217284		
15224372	LOC100155338; LOC102165489	
15229515	LOC100156086; LOC100153281; LOC100624226	
15232410	JMJD1C	
15242729	HMGCS1	
15244515	IL6ST	
15249569	SDC4	
15251413	IGFBP1	
15252889	IFRD1	
15255350	SPTY2D1	
15263008	WEE1	
15266362	STARD4	
15273542	CLDN4	
15279379	CEBPD	
15290288		
15313708		
15314768	IER3	
15317247	NFKBIA	
15324225	SGMS2	
15331932	NAMPT	
15333661		
15334935		

15341205		
15348411		

*** Note: Many of the genes were not traced to any particular locus. The probes that lacked a gene assignment and remained annotated with transcript IDs (NCBI/RefSeq or Ensembl ID), sequence ID (GenBank), or genomic coordinates (GenScan) were filtered out from this set.*

Figure S4: Correlation of markers of tissue injury (amylase and Lactate dehydrogenase/LDH) with serum IL-6 and TNF alpha levels at similar time points after graft reperfusion

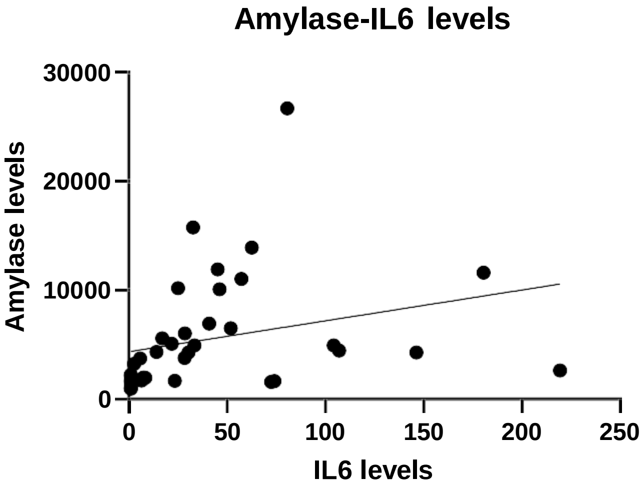

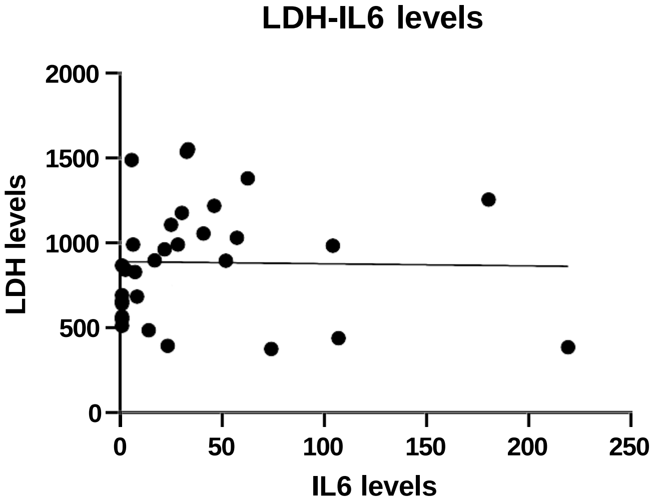

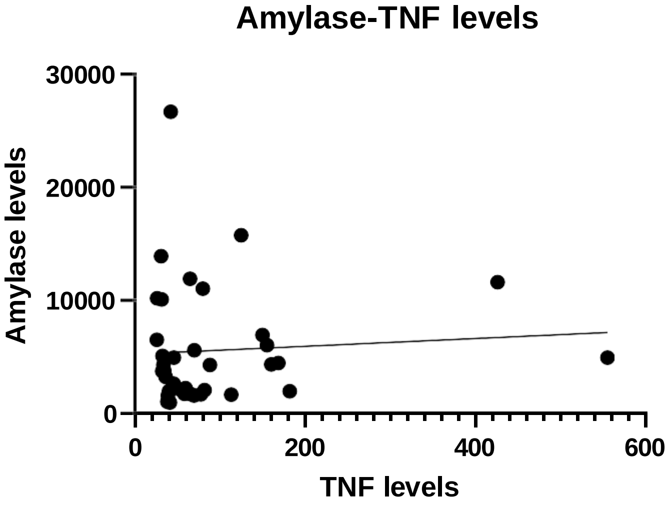

*** Note: Linear regression analysis at similar time points after graft reperfusion. X axis: Serum levels of amylase/LDH and Y axis: serum levels of Il-6/TNF alpha. Amylase-IL-6 correlation: R=0.32 (p=0.67); LDH-IL-6 correlation: R=0.11 (p=0.86); Amylase-TNF alpha correlation: R=0.13 (p=0.52).*
